# Large Unilateral Adrenal Mass with Surrounding Brown Fat: A Case Report

**DOI:** 10.7759/cureus.1552

**Published:** 2017-08-09

**Authors:** Gabriel O Ologun, Zinal M Patel, Navpreet K Rana, Andrew Trecartin, Alice Shen, Douglas Trostle, David Bertsch

**Affiliations:** 1 General Surgery, Guthrie Clinic/Robert Packer Hospital; 2 Medicine, Winthrop University Hospital; 3 Surgical Oncology, Guthrie Clinic/Robert Packer Hospital

**Keywords:** pheochromocytoma, adrenal mass, brown fat

## Abstract

Pheochromocytomas are rare tumors derived from chromaffin cells located in the adrenal and extra adrenal tissues. Pheochromocytomas are diagnosed biochemically and localized using different imaging modalities. The definitive management is surgical resection. Brown adipose tissues are normally present during fetal development, with regression over time. Brown adipose tissues are thermogenic and usually located in the neck, mediastinum, and retroperitoneum. Here, we report a case of a unilateral pheochromocytoma surrounded by brown fat. The abnormal stimulation of brown fat noted on positive emission tomography scan (PET) resolved after the pheochromocytoma was resected.

## Introduction

Adipose tissue is usually classified as white adipose tissue (WAT) or brown adipose tissue (BAT). WAT is the major energy storing tissue while BAT is normally present in fetuses and mediates non-shivering thermogenesis [1]. Norepinephrine has a regulatory function in BAT. Catecholamine stimulation increases the number of brown fat cells, stimulates lipolysis and glucose transport, and uncouples protein-1 expression in brown fat cells resulting in heat production [2-3].

Pheochromocytoma (Pheo) is an uncommon neoplasm that is derived from chromaffin cells. The annual incidence of pheo is about two million and the prevalence in the population is 1:6500 [4]. In approximately 15% to 25%, it arises in the extra adrenal chromaffin tissue, such as the paravertebral ganglia, urinary bladder, posterior mediastinum, and organ of Zuckerkandl [5]. Signs and symptoms of pheo include episodic headaches, sweating, palpitations, anxiety, sustained or paroxysmal hypertension and hyperglycemia [4]. Pheo is diagnosed biochemically and localized using different imaging modalities. The definitive management is surgical resection. Here, we report a case of a unilateral pheo surrounded by brown fat, with imaging depiction of the down regulation of the BAT during the course of treatment. Informed consent was obtained for the case report, images, and for publication.

## Case presentation

**Figure 1:**
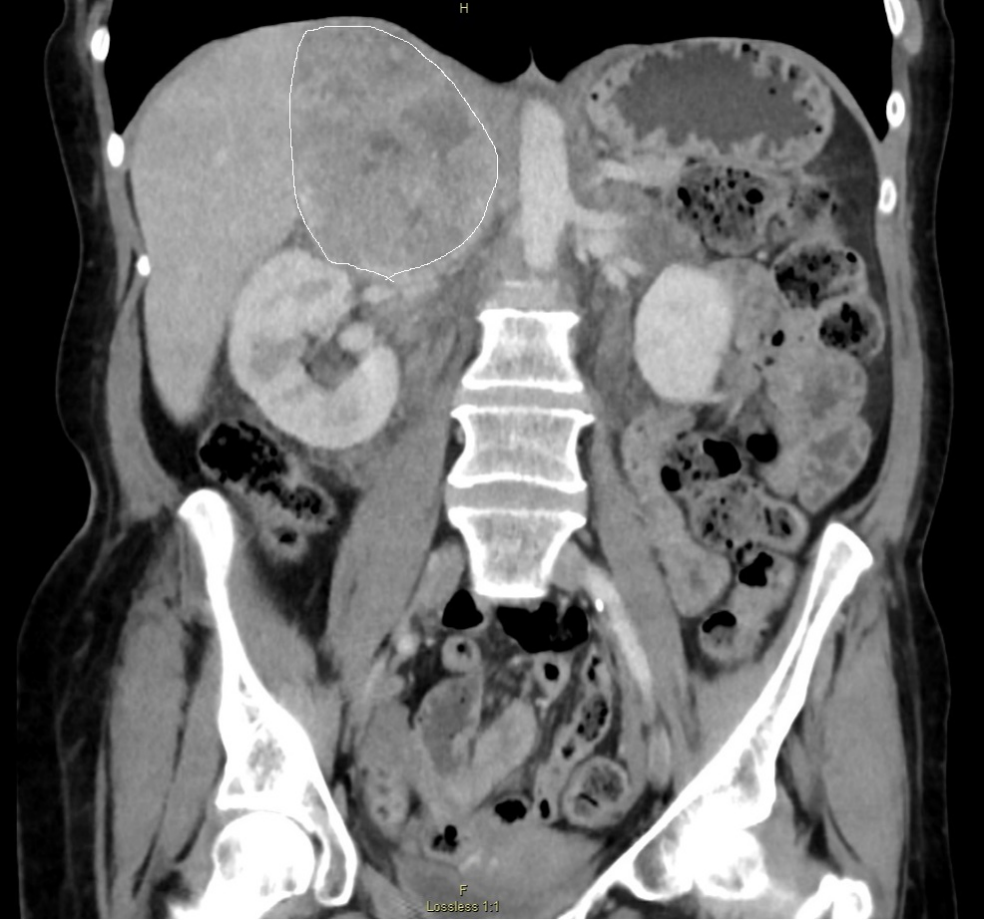
A computed tomography (CT) scan (coronal view) showing a 11.5 cm mass with compression of the vena cava and displacement of the right kidney. The mass is outlined.

A 46-year-old female with an eight-year history of hypertension was referred to our surgical outpatient clinic upon detection of a large abdominal mass noted on a computed tomography (CT) scan of her abdomen and pelvis done during workup for nausea and vomiting. Her symproms also included 35 lb weight loss, palpitations, chest heaviness, sweating, facial flushing, flushing of skin, fatigue with slight exertion, and intermittent headaches over a period of three months. She is a current smoker. Physical examination revealed hypertension and sinus tachycardia, but no facial flushing. There was a flushed look to the forearms, hands, and pretibial lower extremities from the distal third of the lower extremities bilaterally. The abdomen was soft and nontender without masses; the rest of the exam was unremarkable. The CT scan revealed a large right adrenal mass measuring 11.5 x 6 x 9 cm with compression of the vena cava and displacement of the right kidney (Figure 1).

**Figure 2:**
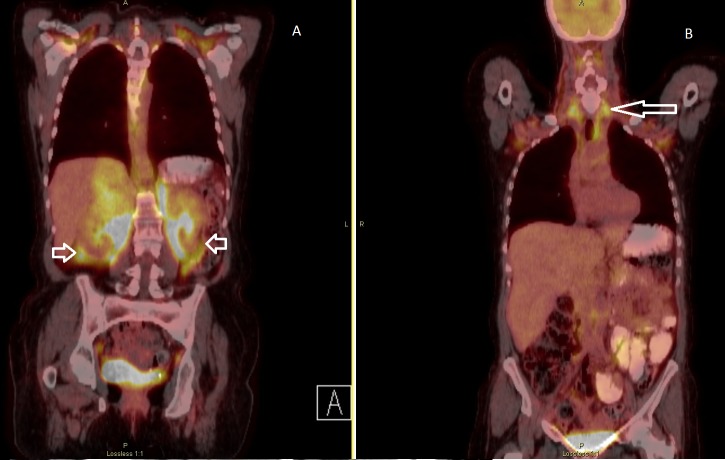
A positive emission tomography (PET) scan combined with computed tomography (CT) (PET/CT) (March 2016) reveals heterogenous moderate fluorodeoxyglucose (FDG) uptake in the large right adrenal gland lesion, with areas of central photopenia, which likely correspond to central necrosis. The extension of the abnormal FDG uptake to involve the para-aortic and aortocaval regions is noted (short arrows, Figure 2A). Also noted is the usual configuration of extensive brown fat uptake of FDG in the neck and thorax (long arrow, Figure 2B).

A positive emission tomography scan combined with CT (PET/CT) in March 2016 revealed heterogenous moderate fluorodeoxyglucose (FDG) uptake in the large right adrenal lesion. There was intense FDG uptake localizing to the unusual and abnormal nodularity and low soft tissue densities surrounding both kidneys, with a standardized uptake value (SUV) of 10.2 (Figure 2).

Biochemical investigations revealed an elevation in serum total metanephrine greater than 13,000 pg/mL, normetanephrine greater than 13,000 pg/mL, plasma renin 175 ng/dL/h, and cortisol of 2.7 ug/dL (ref range 4.5-22.7 ug/dL) after dexamethasone suppression (Table 1).

**Table 1:** Biochemical investigations confirming the pheochromocytoma diagnosis.

	Patient values	Ref Range	Units
Test (Fractionated Plasma)			
Testosterone Total	7	2 - 45	pg/mL
Testosterone, Free	0.7	0.1 - 6.4	pg/mL
Metanephrine	250	<=57	pg/mL
Metanephrine Total	13290	<= 205	pg/mL
Normetanephrine	13040	<=148	pg/mL
Aldosterone, Serum	5	<= 28	ng/dL
Renin Activity	175.94	0.25 - 5.82	ng/dL/h
Test (24-hour Urine Chemistry)			
Aldosterone 24 hr	2	2.3 - 21.0	mcg/24 h
Testosterone Total	7	2 - 45	pg/mL
Testosterone, Free	0.7	0.1 - 6.4	pg/mL

**Figure 3:**
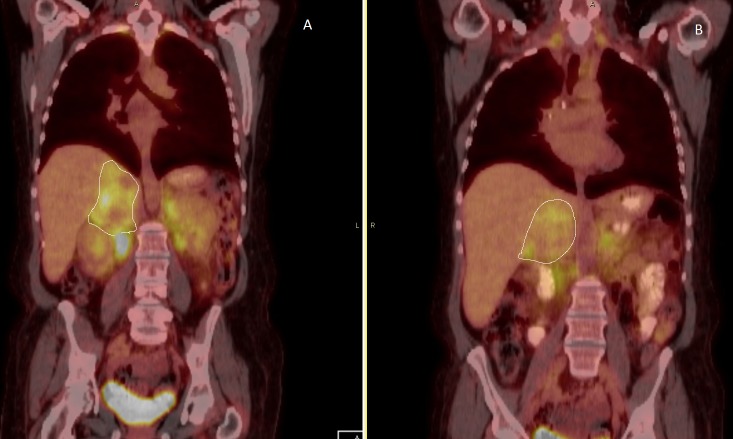
A PET/CT scan (April 2016) after adequate alpha and beta blockade revealed the stable size of the large heterogeneous right adrenal mass (outlined in Figures 3A-3B) with moderate FDG uptake with areas of central photopenia, which likely correspond to central necrosis. There is intense FDG uptake in the perinephric space with a maximum SUV of 7.4 compared to 10.2 in the previous study. PET/CT - Positive emission tomography/computed tomography, FDG - fluorodeoxyglucose, SUV - standardized uptake value.

The patient was presented at our multidisciplinary tumor board. Upon further review of the imaging studies, the finding on the PET scan suggested the possibility of diffuse brown fat development and evidence of hypermetabolism, symmetric in the neck, retroperitoneum, and inguinal regions. Overall, this appearance was consistent with pheochromocytoma with brown fat stimulation and less likely suggestive of metastatic pheochromocytoma. Preoperatively, adequate catecholamine blockade was achieved using alpha adrenergic blocker and beta blockage. Her symptoms improved. A repeat PET/CT in April 2016 after adequate alpha and beta blockade revealed the stable size of the large heterogeneous right adrenal mass with moderate FDG uptake with areas of central photopenia, which likely correspond to central necrosis. No significant retroperitoneal or intra-abdominal lymphadenopathy was noted (Figure 3).

**Figure 4:**
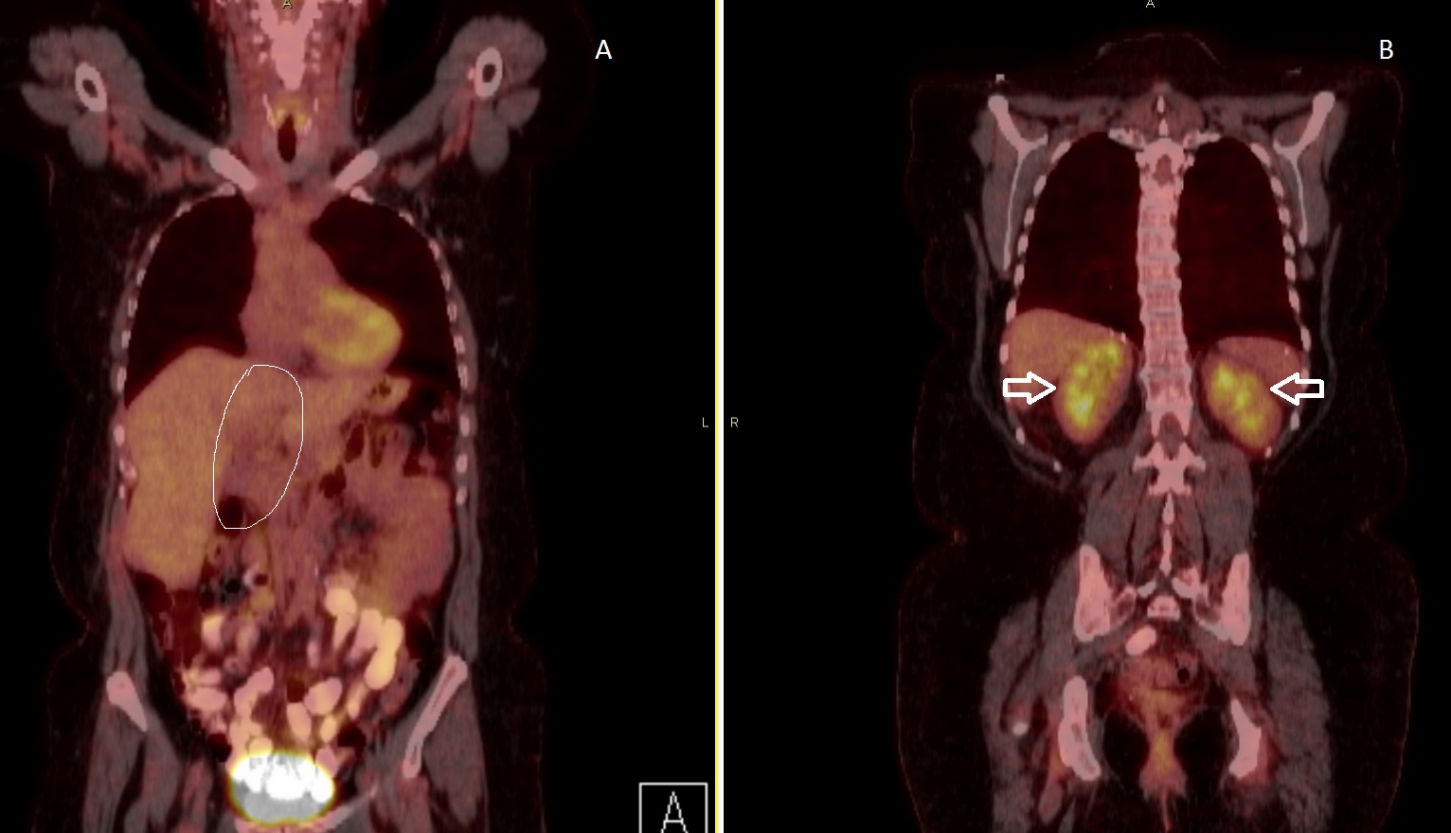
A PET/CT in October 2016 revealed no focal abnormality in the resection bed (outlined in Figure 4A), no gross evidence of metastatic disease, no significant retroperitoneal or intra-abdominal lymphadenopathy. Normal uptake was noted in bilateral kidneys (short arrows in Figure 4B). PET/CT - Positive emission tomography/computed tomography.

The patient was taken to the operating room in May 2016. An open right adrenalectomy was performed through right subcostal incision with extension up the midline to the xiphoid. A large adrenal mass was noted with significant periadrenal gland fibrosis. The mass was resected en bloc with a portion of the right hemidiaphragm. Intraoperatively, there were significant fluctuations in the patient’s blood pressure, which was appropriately managed by the anesthesia team. There were no other significant surgical complications, and the patient made an uneventful recovery. She was discharged on postoperative day 10. Five months later, the patient remained stable and had no symptom to suggest recurrence. A PET/CT scan in October 2016 revealed no focal abnormality in the resection bed, no gross evidence of metastatic disease, no significant retroperitoneal or intra-abdominal lymphadenopathy (Figure 4). Given the risk of recurrence, long-term follow-up with CT scan of the chest, abdomen, and pelvis, and serum metanephrine studies is recommended.

Pathological examination of the surgical specimen revealed an ovoid soft tissue mass measuring 12 x 9.3 x 5 cm and weighed 270 g. The capsule appeared intact. Sectioning revealed a red-brown-yellow, soft and lobulated cut surface with fibrous septae and focal areas of hemorrhagic degeneration. Immunohistochemistry revealed tumor cells positive for chromogranin and synaptophysin. The final pathologic diagnosis was pheo of the right adrenal gland with lymphovascular invasion and no perineural invasion; inked margin was uninvolved.

## Discussion

Pheochromocytoma is an uncommon neoplasm that is derived from chromaffin cells. The annual incidence of pheo is about two million and the prevalence in the population is 1:6500 [4]. It occurs in about 0.05% of patients with sustained hypertension, accounting for only about 50% of the patients with pheo, as only about half of these patients will have paroxysmal hypertension or normotension [4, 6]. In approximately 75% to 85% of adults with pheo, it arises in the adrenal medulla, in 15% to 25%, it arises in the extra adrenal chromaffin tissue, such as the paravertebral ganglia, the urinary bladder, the posterior mediastinum, and the organ of Zuckerkandl [5]. Multiple endocrine neoplasia (MEN) IIA and MEN IIB, neurofibromatosis, and Von Hippel-Lindau disease can have synchronous, metachronous, or extra-adrenal involvement. Today, extra-adrenal, prevalence of malignancy, and bilaterality are directly dependent on the underlying genetic mutation [7]. The signs and symptoms of pheo include episodic headaches, sweating, palpitations, anxiety, sustained or paroxysmal hypertension, and hyperglycemia [4]. The physical exam is usually unremarkable. The biochemical diagnosis of pheo is made by demonstrating elevated plasma metanephrine and/or normetanephrine, or 24-hour urine excretion of catecholamine and their metabolites such as metanephrines and vanillylmandelic acid [4]. Radiographic studies are used to demonstrate the presence of an adrenal mass. CT scan with and without intravenous contrast and magnetic resonance imaging (MRI) are the two most commonly used imaging modalities in the initial evaluation of pheo. CT scans are highly sensitive (88%-100%) but lack specificity [7]. MRIs can be useful in distinguishing pheochromocytoma from an adenoma. Pheo has a characteristic high intensity on T2-weighted images compared to adenoma. Functional imaging, including 123 I-metaiodobenzylguanidine (MIBG), 111-In-pentetreotide (octreotide scan), and PET/CT using fluorodeoxyglucose (FDG) and other radiolabeled agents, is also used for the localization of pheo. Metaiodobenzylguanidine with single-photon emission computed tomography (MIBG-SPECT) is a guanethidine analog resembling norepinephrine that is taken up and concentrated in sympatho-adrenergic tissue [7]. The management of benign and malignant pheochromocytoma is surgical excision. Malignancy is demonstrated by local invasion. Preoperative preparation includes the administration of an alpha-adrenergic blocker to control hypertension and permit re-expansion of intravascular volume. Phenoxybenzamine, 10 mg orally twice a day, is initiated as soon as the diagnosis is made and it is increased until the desired effect (postural hypotension) or prohibitive side effects are encountered. Prazosin, terazosin, and doxazosin have been used as alternatives to phenoxybenzamine. These drugs are short-acting alpha-1 antagonists [7]. Beta-adrenergic blockade (for example propranolol) may be added if reflex tachycardia or arrhythmias develop, but only after alpha blockade is optimized. In patients with sporadic, unilateral pheochromocytoma localized by preoperative imaging studies, adrenalectomy may be performed by anterior or posterior open approach or by laparoscopic adrenalectomy [4, 7]. The operative approach for familial pheochromocytomas is the exploration of the adrenal glands, the preaortic and paravertebral areas, and the organ of Zuckerkandl through a midline or bilateral subcostal incision. Intravenous phentolamine, sodium nitroprusside, propranolol, and vasopressors should be immediately available in the operating room, as intraoperative labile hypotension or hypertension can occur during the resection of the pheochromocytoma. This can be avoided by minimal manipulation of the tumor. An experienced anesthesiologist and euvolemia are essential. In the immediate postoperative period, watch for hypoglycemia due to increased insulin and possible congestive heart failure due to cardiomyopathy. Following pheochromocytoma excision, about 15% will develop recurrence [8]. Long-term follow-up with regular interval office visits, catecholamine checks, and imaging studies is necessary. Adipose tissue is usually classified as white adipose tissue (WAT) or brown adipose tissue (BAT). WAT is the major energy storing tissue while BAT mediates non-shivering thermogenesis [1]. BAT is normally present in fetuses and diminishes in adults, accounting for approximately 1% of the total body weight [2]. Adrenergic stimulation induces an increase in BAT, known as “browning”, a phenomenon important due to its potential use in the management of obesity and obesity-related disorders [3]. BAT has more sympathetic adrenergic innervation than white adipose tissue [2]. Norepinephrine has a regulatory function in BAT. Catecholamine stimulation increases the number of brown fat cells, stimulates lipolysis and glucose transport, and uncouples protein-1 expression in brown fat cells resulting in heat production [2-3]. Remnants of BAT in adults are usually found in the neck, mediastinum, axilla, retroperitoneum, and abdominal wall [9].

## Conclusions

In this report, we presented a case of a 46-year-old female with a large pheo that was surrounded by brown fat. The diagnosis of pheo was made by studying plasma and urine metanephrine levels while imaging studies were used to localize the mass. The management consisted of providing alpha blocker followed by surgical resection of the mass. Our patient remained stable and disease free five months after resection. The abnormal stimulation of brown fat noted on the PET scan resolved after the pheochromocytoma was resected.
